# Generational issues in linking family farming production, traditional food in diet, physical activity and obesity in Pacific Islands countries and territories: the case of the Melanesian population on Lifou Island

**DOI:** 10.12688/openreseurope.13705.2

**Published:** 2022-10-11

**Authors:** Olivier Galy, Stéphane Frayon, Marco Goldin, Paul Zongo, Guillaume Wattelez, Sonny Lameta, Alan Quartermain, Jean Marie Fotsing, Séverine Bouard

**Affiliations:** 1 Interdisciplinary Laboratory for Research in Education, EA 7483, University of New Caledonia, Noumea, New Caledonia; 2 TERAU: Territoires, acteurs et usages, Institut Agronomique néo-Calédonien (IAC), Pouembout, New Caledonia; 3 School of Agriculture and Food Technology, The University South Pacific, Samoa Campus, Fiji; 4 School of Science & Technology, The University of Goroka, Goroka, 1078, Papua New Guinea; 5 ISEA, University of New Caledonia, Noumea, New Caledonia, 98800, New Caledonia

**Keywords:** agriculture, nutrition, physical activity, lifestyle, health, overweight, traditional knowledge

## Abstract

In the Melanesian culture, traditional activities are organized around family farming, although the lifestyle transition taking place over the last several decades has led to imbalances in diet and physical activity, with both leading to obesity. The aim of this interdisciplinary study was to understand the links between family farming (produced, exchanged, sold, and consumed food), diet (focused on produced, hunted, and caught food), physical activity (sedentary, light, and moderate-to-vigorous physical activity) and obesity in Melanesian Lifou Island families (parents and children). Forty families, including 142 adults and children, completed individual food frequency questionnaires, wore tri-axial accelerometers for seven continuous days, and had weight and height measured with a bio-impedance device. A family farming questionnaire was conducted at the household level concerning family farming practices and sociodemographic variables. Multinomial regression analyses and logistic regression models were used to analyze the data. Results showed that family farming production brings a modest contribution to diet and active lifestyles for the family farmers of Lifou Island. The drivers for obesity in these tribal communities were linked to diet in the adults, whereas parental socioeconomic status and moderate-to-vigorous physical activity were the main factors associated to being overweight and obesity in children. These differences in lifestyle behaviors within families suggest a transition in cultural practices at the intergenerational level. Future directions should consider seasonality and a more in-depth analysis of diet including macro- and micro- nutrients to acquire more accurate information on the intergenerational transition in cultural practices and its consequences on health outcomes in the Pacific region.

## Plain language summary

In the Pacific region, populations have gained faster access to modern lifestyles in the past few decades, causing fundamental changes in the way people move about and eat (including food choices, physical activity, and sedentary time) and a dramatic increase in noncommunicable diseases. The Melanesian population of New Caledonia is particularly exposed to the changing life while contemporary family subsistence activities have been maintained despite the growth in development hubs. It is also important to understand the place of family farming and its influence on health and the possible changes of behavior between parent and children in the Melanesian population. This pilot case study implicated forty Melanesian Lifou Island families (parents and children) and showed that family farming production brings a modest contribution to diet and active lifestyles for the family farmers of Lifou Island and suggest a transition in cultural practices between generations.

## Introduction

Family farming is one of the pillars of cultures all over the world and is at the heart of the United Nations 2019-2028 strategy, Decade of Family Farming ( http://www.fao.org/family-farming/detail/en/c/1195619/), which highlights the crucial role of family farming in eradicating hunger and shaping the future of food. In the Pacific region, populations have gained faster access to modern lifestyles in the past few decades, causing fundamental changes in the way people move about and eat (including food choices, physical activity, and sedentary time) and a dramatic increase in noncommunicable diseases ^1– 3^. A recent study from the Pacific region nevertheless showed that individuals living on remote atolls had the most traditional lifestyles and the healthiest lifestyles ^4^. Yet overall, food consumption has shifted from a traditional diet of mostly fresh fish, vegetables, and tubers to a modern diet that includes canned meat or fish, oil, sugar, rice, and processed foods ^5^. The energy expenditure of family farmers, which is associated with family farming production and nutritional outcomes, is relatively unknown ^6^, especially in the Pacific region ^7^. The influence of energy expenditure at individual and household levels is therefore still not well understood and remains a “black box” for researchers ^6^. However, interdisciplinary approaches based on the agricultural, food, exercise, and health sciences might comprise a set of comprehensive tools for opening this “black box” ^6, 8^. This has major importance for societies in the Pacific region where family farming is one of the pillars of culture and the traditional lifestyle.

The Melanesian population of New Caledonia is particularly exposed to the transitioning environment. New Caledonia, a French territory in the Pacific, is fast approaching the socioeconomic level of Western countries ^9^ because of French financial transfers, the recent growth in nickel mining, and local transformation to ferronickel in the past few decades.

In Melanesian societies, contemporary family subsistence activities have been maintained despite the growth in development hubs ^10^, increases in education levels, and improvements in material conditions, as these have not been systematically synonymous with a decline in agricultural activities, hunting, and fishing ^11, 12^. However, the food environment is changing how people access, prepare, and consume food ^13^. Today, an increasing demand for packaged imported foods, such as canned meats, instant noodles, cereals, rice, and sugar-sweetened beverages (SSBs), with the subsequently decreased consumption of locally produced plants and animals, has led to a heightened vulnerability to food insecurity ^14^. Emergent food environments in low- and middle-income countries (LMICs) have created conditions where “people often choose to eat lower-cost, less-healthy, more energy-dense foods, choices that can lead to people becoming overweight and obese as their means to access healthy food diminish” ^15^. In New Caledonia, choices are even more constrained in a context of high living costs linked to the narrowness of the economy and its oligopolistic structure ^16^. The hyper-insularity of Lifou has further amplified the transport costs and the difficulties of being supplied with healthy products at affordable prices. Recent studies on adolescents have reported specific dietary patterns including high consumption of meat, fast food, fruits and vegetables, and sweets, with few dairy products ^17^; low consumption of energetic foods ^18^; and high consumption of highly processed drinks like SSBs ^19^ and energy drinks ^20^. This has been accompanied by breakfast skipping ^21^ and relatively low physical activity ^22, 23^. These changes might reflect an intergenerational nutrition transition within families and plays a major role in the high levels of overweight and obesity in Melanesian families, especially those living in rural areas on customary lands.

Yet, the Melanesian population has continued to maintain a traditional Pacific tribal lifestyle, especially on the Loyalty Islands, which are characterized by traditional family farming, fishing, and cultural activities that follow tribal customs (e.g., house construction, weddings, and mourning) ^24^ and generally require vigorous daily physical activity. One of these Loyalty Islands, Lifou, is inhabited mainly by Melanesian people living on customary lands and is 40 minutes by plane from Noumea, the capital of New Caledonia. Lifou’s small-scale family farming predominates in the organization of the ancestral agricultural system and is one of the pillars of the traditional cultural practices that contribute directly and indirectly to the sustainability of community living on customary lands. Indeed, family farming contributes directly to the economy (by ensuring food security and selling on local markets), socialization (strengthening social and family ties through daily donations of plant production and mutual aid in the fields) and culture (maintaining customary traditions and donations) of the families within tribes ^10^.

To sum up, New Caledonia today is in the earlier stages of a transition already observed in countries like the USA and those in the Pacific region (Australia, Fiji, and New Zealand). “It is a nutrition transition, in which traditional food environments based on farming and hunting are being replaced by those where foods are designed by chemists and food technologists to appeal to human appetites, then manufactured and shipped to every corner of the earth, always with the same consequences: skills associated with traditional feeding habits are lost, and obesity and associated diseases increase” ^25^. However, almost nothing is known about the agricultural practices of families (parents and children) living on customary lands and their influence on daily diet, physical activity, and health outcomes. For this 5uestin, we assumed that Lifou Island would offer a valuable case study of what we might observe in advanced transitioning Pacific Island countries and territories (PICTs), which would then help in setting policies for future actions. We therefore hypothesized that, in the current socioeconomic context of transition, practices linked to family farming like eating food from the sea, bush and garden (tubers, fruits, vegetables, hunted animals and drinking water) and the related physical activities (on the crop plot) are transitioning across generations and are not strong enough to feed families and counteract an insufficient energy expenditure. As a consequence, they do not play a sufficiently strong role in preventing overweight and obesity that has been shown to be high in the Melanesian population of New Caledonia ^17, 26^.

The aim of this study was to determine the links between family farming (produced, exchanged, sold, and consumed food), diet (focused on produced, hunted, and caught food), physical activities (sedentary, light, and moderate-to-vigorous physical activity) and obesity in Lifou Island families (parents and children).

## Methods

### Ethics statement

We obtained informed written consent from all parents for their participation and their children’s participation before their children entered the study. The research met the legal requirements and the Declaration of Helsinki, and the protocol ^27^ was approved by the Consultative Ethics Committee of New Caledonia: CCE 2018-06 01.

### Data Collection and Participants

**Figure 1.**
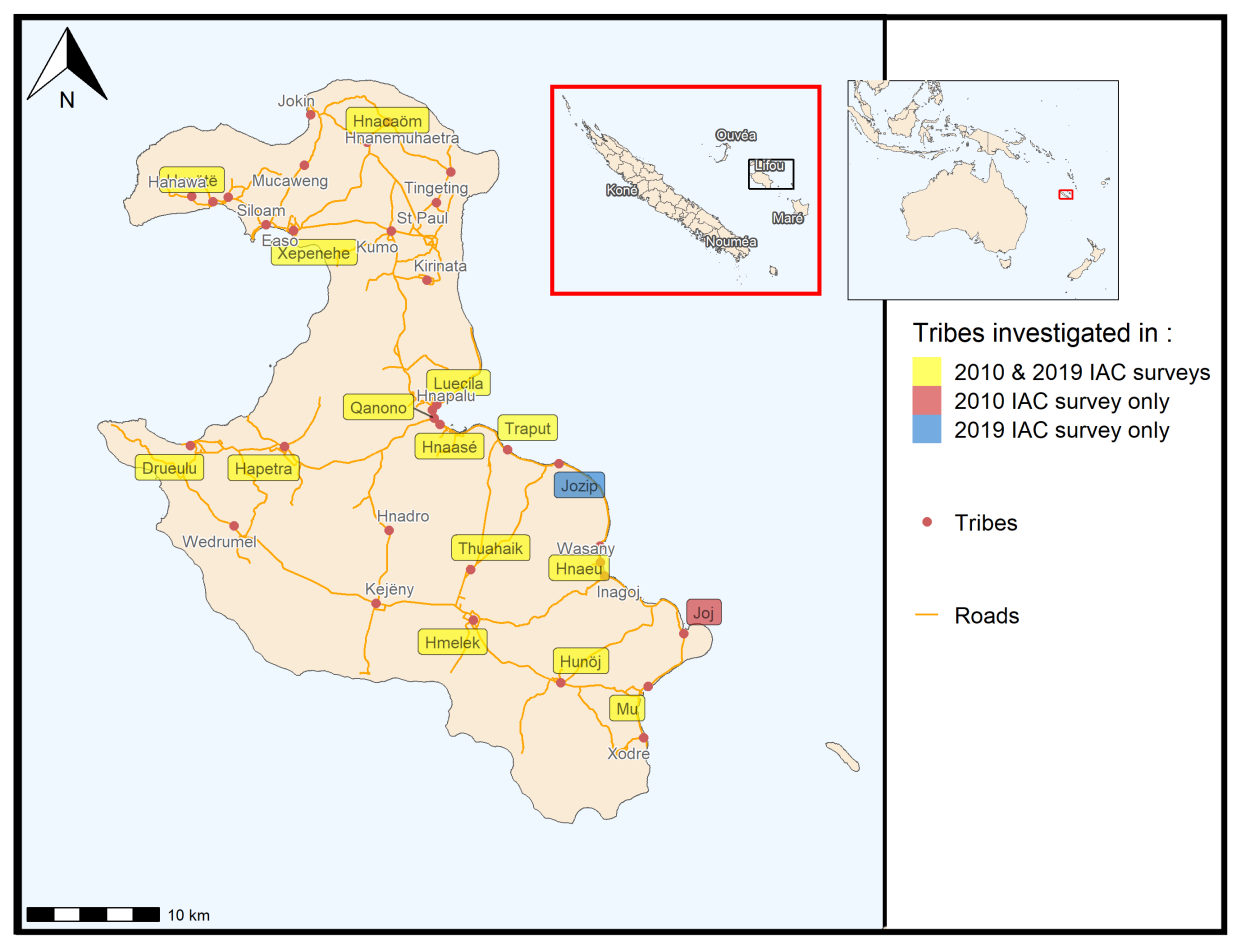
Map of New Caledonia showing the main island, the Loyalty Islands and Lifou Island with the investigation sites in 2010 and 2019.

Our study was conducted on Lifou Island ( Figure 1) in New Caledonia. The eligibility of the participants was to live all year round on Lifou Island, which is a customary land with one or more children in the public secondary school. Customary land is a Melanesian territory that follows both customary and civil society rules. Among these rules, any initative done by civil institutions, here a research program on Lifou families, must be submitted to the customary authorities to get an agreement. The customary authorities of Lifou island (Loyalties Islands Province) were first contacted to get a approval to do the study with the families among the three districts: Wetr, Gaica and Lössi ( Figure 1). Then, the study was proposed to 48 families from 16 tribes, all belonging to the three districts of Lifou Island. Families were selected among the classes (two classes of 25 children per level across four levels: 6 ^ième^ (11–12 years), 5 ^ième^ (12–13 years), 4 ^ième^ (13–14 years), and 3 ^ième^ (14–15 years)) of the public school of Lifou. In each class, six families were randomly selected by grouping all the families in each class into the three districts of Lifou and then randomly selecting two from each district by pulling numbers out of a box. The selected families were then contacted in alphabetic order. This permits us to select six different families among the two classes of each of the four levels of the public school (total potential participating families – 48). Calls and the first visit was done by a member of the research team native from Lifou. At the end of the selection process, 40 families agreed to participate in this study. In each household, all the adults over the age of 18 years old and any children/adolescents aged 10 to 18 years old were invited to participate. 

We gathered data from June 2019 to July 2019 from 142 children and adults, aged from 11 years old to adulthood. Data were collected during the simultaneous free time of parents and children (at dusk, at the end of the work and school day, or on the weekend). The questionnaires used in this study with the families during face-to-face interviews can be found in the extended data section ^28^ and were carried out by two researchers who were chosen for the following reasons. The first researcher involved in this stage had previous expertise in family farming survey and the other researcher had expertise in food frequency questionnaires, anthropometrics, and accelerometry. Moreover, the second researcher was native to Lifou Island without any family relationships with any of the participants, but was able to communicate in the native language with participants when necessary. In each family surveyed, children with missing data were then excluded from this study (n=3). Table 1 presents the details of the data variables recorded during the study.

**Table 1.** Description of the variables recorded during the study. Data concern family farming were recorded at the family level and data concerning diet, physical activity via accelerometer, and descriptive variables of health were recorded at the individual level. Variables, tools, methods, and units are detailed, including units.

Theme	Level of observation	Variables	Tools	Methods	Units
**Family farming**	**Members of ** **the household**	Farming-related activities of each member	Questionnaire/ interview	Mixed	N/A
	**Household**	Land use/cultivated area	Questionnaire	Quantitative	Sq meters or acre
		Agricultural equipment, fishing gear	Questionnaire	Quantitative	Local currency
		Crop, livestock production, fisheries, hunting	Questionnaire	Quantitative	Kg
		Destination (auto- consumption, gift, personal consumption)	Questionnaire	Quantitative	Local currency, kg
		Crop production, livestock, hunting and fishing costs	Questionnaire	Quantitative	Local currency, kg
		Monetary incomes	Questionnaire	Quantitative	Local currency
**Diet**	**Members of ** **the household**	Diet	Food frequency qestionnaire	Quantitative	Categories, % of diet
**Physical activity**	**Members of ** **the household**	Physical activity	Wrist Accelerometry	Quantitative	Nature of activities, duration (min), frequency, time (min/day), bouts of activity for sedentary, light, and moderate-to-vigourous physical activity.
**Descriptive ** **variables & ** **health outcome ** **variables**	**Members of ** **the household**	Descriptive variables: Age, gender, education, occupation	Questionnaire	Quantitative	Year, N/A
		Body composition	Bioimpedancemetry/ scale	Quantitative	Kg, Body Fat %, Total Body Water %, Muscle Mass, Physique Rating, Bone Mineral Mass, Basal Metabolic Rate, Metabolic Age, Body Mass Index, Visceral Fat
		Body height	Height gauge/ruler	Quantitative	Cm

### Measures

***Family farming questionnaire.*** Before visiting the family, a member of the team native from Lifou contacted each family by phone to explained that we were trying to understand more about the situation in rural Lifou households and that we would like to do a survey with the head of the household or the person in the household that has the most knowledge about agriculture activities and production. Once we obtained the agreement of the family, a visit was scheduled. Then, the questionnaire was 9uestion at home with the heads of households in order to collect all the data in a single operation. The household themselves identified the household head. Then, we asked if the household head would have an hour to spare to complete the questionnaire ^28^. If the household head was not available, then we asked to speak to some of the adult household members only if these household members felt they could represent the household situation well.

The survey design was deeply inspired by a wide survey conducted in 2011 covering all of New Caledonia ^11, 29, 30^. As the aim was to understand the roles and links of agricultural, fishing, and hunting activities with food practices and diet, the observation unit was the household in order to focus on the traditional social organization and noncontractual family farming with a prevailing nonmarket dimension ^24^. For family farming, the main observation unit was the household, which comprised all the people living on the same residential plot, sharing meals and some of the agricultural work. The structure of the survey was based on the “sustainable rural livelihoods” ^31, 32^ approach with a microeconomic perspective. The multi-thematic questionnaire used in this study was the questionnaire deployed in almost all tribes in New Caledonia in 2011 ^11, 30^ and made it possible to quantitatively measure the agricultural volumes produced by the tribal population in 2018, as well as the distribution of these volumes between self-consumption, daily gifts, ceremonial gifts, and market sales. The first goal of this survey, referenced as the 2019 dataset in this paper ^28^, was to define the household composition (number of persons, education, land access and ownership, monetary incomes). The second was to quantify the agricultural production (plants, cattle, hunting, fishing, and food gathering) and the allocation of these productions in the eating and sharing (daily and ritual exchanges) habits of the families. As the majority of the households do not weigh their harvests and keep no accounting records, the questionnaire relied on daily practices, taking into account the wide range of tools used for harvesting purposes (plastic bags, tote bags, palm-leaf baskets, etc.) and the harvesting practices (weekly, monthly, during periods of traditional customs). These quantities were then converted into conventional units of measure using charts created specifically for the study based on measurement results taken from a sample of units used locally.

The production of each household was broken down according to: (1) plant production, including the production of tubers (taro, yam, cassava, etc.) from the tuber category in the data file, the production of fruits and vegetables (avocado, star fruit, lemon, salad, etc.) from the categories of fruit trees, bananas, dessert bananas, poingo bananas, coconuts, field fruits, and vegetables in the data file ^28^, and the total crop production; (2) animal production (pigs, poultry, etc.); 3) hunting production (flying foxes, wild pigs, pigeons, etc.); and 4) fishing production (sea fishing, shellfish and crabs, etc.). For each type of production, a distinction was made according to the finality of production: sale, personal consumption, exchange, or total production.

 In order to accurately acompare individual data, we converted “farming data” according to the number of people in the household. The proportion of adults, children, and adolescents was not taken into account in this calculation. This means that for a household comprising n people, the announced consumption was divided by n. For example, household one had six members and the consumption of tubers is 139.9 kg. We therefore associated this household with a consumption of approximately 23.3 kg of tubers/person/year s*. Finally, the ratio computation was mainly performed in order to avoid an imbalance in production and consumption of production between individuals belonging to large families and those belonging to small families.

***Food frequency questionnaire (FFQ).*** The short FFQ was adapted from the validated version of the FFQ for Aboriginal and Torres Strait Islanders by Gwynn *et al.,* ^33^, in the absence of a validated FFQ for New Caledonia. Minor modifications were made by the research team to include foods identified as important in the diet of Melanesians ^5^ and described elsewhere ^18^ and in the extended data section ^28^. We then classified food in the ‘traditional food’ category, and the ‘limited food’ and ‘limited drinks’ categories. The traditional food category helped us estimate the proportions of tubers, fruits and vegetables, meat, and fish eaten as well as water drank at home. We focused on the consumption of traditional food like tubers, fruits and vegetables, and fish, and of limited food and drinks classified as follows by the Pacific Community guidelines ^5^: foods to limit (butter + breakfast cereals + canned meat + cold cuts + noodle soup + fries + chips + hamburger + sweets + cookies) and drinks to limit (SSBs). For the analyses, we categorized individual consumption as follows: low tuber consumption: <1 unit/week, medium tuber consumption: 1 to ≤5 units/week, high tuber consumption: >5 units/week; low fruit and vegetable consumption: ≤14 units/week, medium fruit and vegetable consumption: >14 to ≤35 units/week, high fruit and vegetable consumption: >35 units/week; low fish consumption: ≤1 unit/week, medium fish consumption: >1 to ≤3 units/week, high fish consumption: >3 units/week; low limited food consumption: ≤2 units/week, medium limited food consumption: >2 to ≤5 units/week, high limited food consumption: >5 units/week; and low limited drinks consumption: ≤1 unit/week; medium limited drinks consumption: >1 to ≤ 10 units/week, high limited drinks consumption: >10 units/week.

***Physical activity.*** The weekly physical activity levels of each family member were assessed via GENEActiv activity trackers positioned on the nondominant wrist for seven days starting on the day of the FFQ survey. This weekly physical activity level was not detailed per activity (i.e.: walking, hunting, garden activity). The datasets from these trackers contained 60-Hz 3-dimensional accelerometer data. Raw data were processed into one second epoch signal vector magnitude data points of daytime activity and were then categorized into physical activity (PA) levels: sedentary, light, and moderate-to-vigorous PA (MVPA) for each second. PA was analyzed according to the WHO recommendations ^34^ and a more in-depth analysis for PA patterns in the adults was conducted using Diaz and Yacef’s method ^35^. For children, we used Phillips’s cut points ^36^. We focused on bouts of sedentary activity, light activity, or MVPA occurring with a minimum duration. A bout was defined as a continuous episode of PA at a specific range of intensity, and the length of a bout was the number of seconds spent at that intensity during that episode. Thresholds for sedentary bouts (60 seconds minimum duration), light bouts (10 seconds minimum duration), and MVPA (three seconds minimum duration) were based on the literature ^35^.

***Anthropometry.*** A trained researcher who is a native from Lifou Island without any family relationships with any of the participants and with an expertise in sports sciences collected the anthropometric data in the homes as outlined in the data collection section. A portable stadiometer (Leicester Tanita HR 001, Tanita Corporation, Tokyo, Japan) measured height to the nearest 0.1 cm. Weight was assessed to the nearest 0.1 kg using a 11uestionnair scale (Tanita HA 503, Tanita Corporation, Tokyo, Japan), with the participants wearing light clothing. From these measurements, body mass index (BMI) was calculated in adults as follows: BMI = weight [kg]/([height [m]) ^2^. Normal weight was considered a BMI≤25; overweight was a BMI>25 and obesity was a BMI>30. We used the International Obesity Task Force (IOTF) criteria for children to define adolescents as thin (underweight), normal weight, overweight, or obese ^37^. The IOTF criteria provide BMI cut-offs for weight status based on BMI values according to age and sex ^37^.

***Sociodemographic status.*** Ethnicity was self-reported by each family member and categorized as recommended in the report on New Caledonia (INSERM, 2008) from the Institut National de la Santé et de la Recherche Médicale (INSERM; National Institute of Health and Medical Research). Three socioeconomic (SES) categories were determined based on the National statistics socio-economic classification: managerial and professional occupations (high), intermediate occupations (medium), and routine and manual occupations (low).

### Statistics

We first tested correlations between the consumption of traditional foods (tuber, fruits and vegetables, fish) and processed foods (limited food and SSBs), and family farming activities, namely, the garden surface per family member, as well as the tuber, fruit and vegetable, fish, hunting and animal productions per family member. Multinomial regression analyses were used to study these relations and the effect plots of the significant variables are presented. Secondly, we examined the relationships between agriculture and PA (quantity of MVPA, light activity, sedentary time, and following WHO recommendations) through linear regressions for the continuous explained factors and logistic regressions for the binary explained factors. Lastly, relationships of agriculture, nutrition and PA with weight status were analyzed by consecutive logistic regressions with factor selections. The final models are presented. All analyses were adjusted by socioeconomic factors (sex, age, size of family, and the highest school level of the household head) in both adults and children.

Analyses were conducted using R 3.5.1. ^38^, with an accepted type I error probability set at α=0.05. The multinomial regressions were computed thanks to the *multinom* function from the *nnet* package ^39^. The *effects* package was used to draw the effect plots ^40– 42^. The linear regressions were computed with the *lm* function, the logistic regressions with the *glm* function, and the factor selection with the *step* function.

## Results

### Sample characteristics

In the traditional foods of the adult group, vegetables, legumes, fruits, and tubers were present in the daily adult diets and were largely above the recommendations for vegetables, legumes, and fruits, while a low tuber consumption was observed. The duration and intensity of PA were largely below the WHO’s international recommendations for both sexes. In addition, more than 85% of the adult group was overweight or obese.

**Figure 2.**
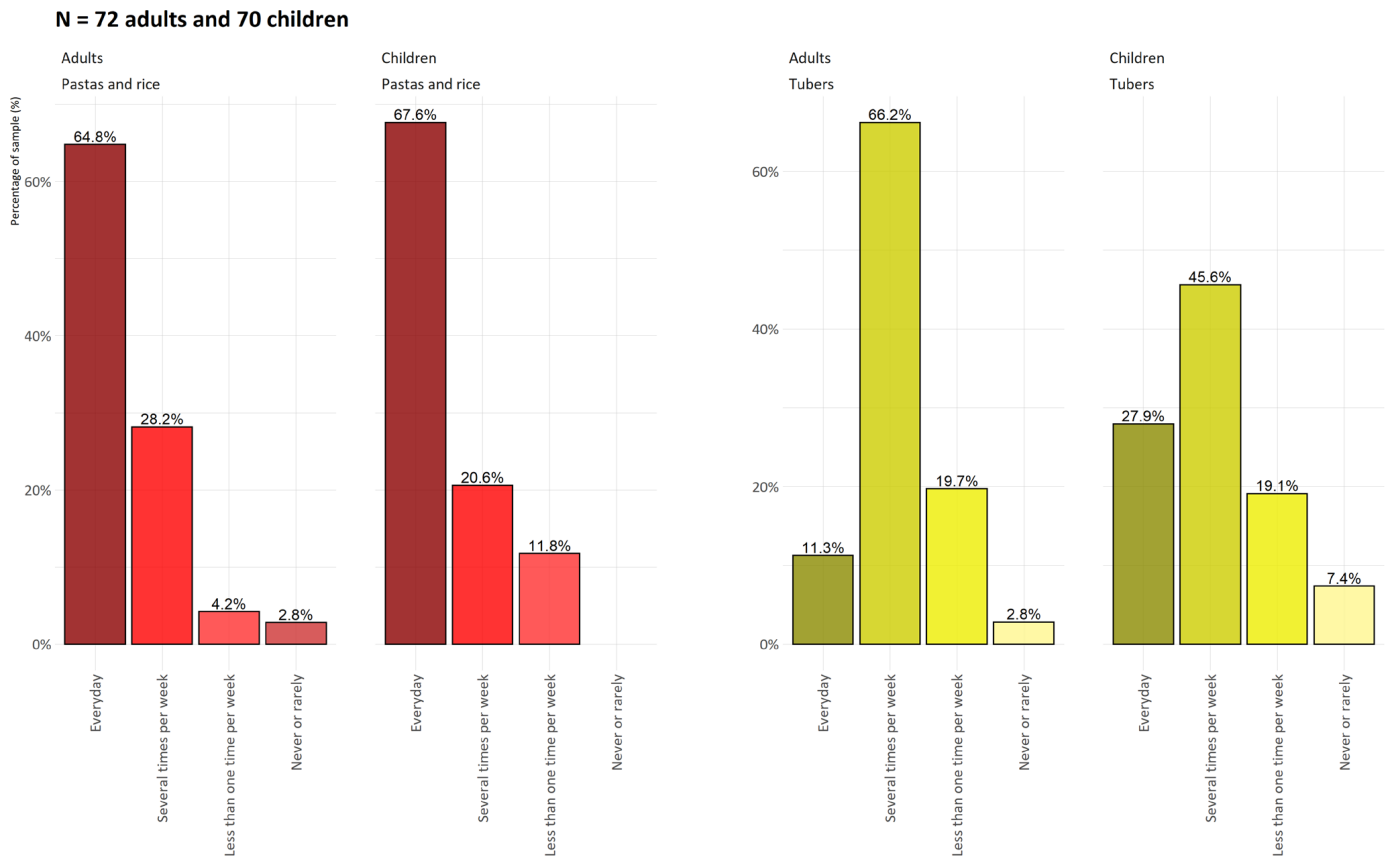
Frequency of consumption of pasta, rice and tubers for adults and children on Lifou Island in 2019.

In the traditional foods of the children’s group, vegetables, legumes, fruits, and tubers were present in their daily diets and largely above the recommendations for vegetables, legumes and fruits, while a low tuber consumption was observed. Figure 2 shows the frequency of consumption in terms of percentage of pasta, rice, and tubers for adults and children. Concerning PA, girls were largely below the international recommendations for MVPA in terms of duration and percentage. We also note that more than 30% of the children’s group was overweight or obese, with more than 25% of the girls overweight and obese.

### Characteristics of households

In comparison with the 2010 national survey conducted in the three provinces and on Lifou, our results appear similar for total crop, animal, and fishery production. With the Wilcoxon rank sum test, we can see differences in the destinations of crop production: between 2010 and 2019, exchanged crop production decreased in favor of consumption. For hunting (wild pigs, green pigeons, and flying foxes), we can see that the two sets of data are statistically different, with fewer total quantities hunted in 2019. This decrease in hunting activity would need to be studied in more detail and verified with a larger sample, but it was also observed on the mainland of the archipelago, among 77 tribal households in the Northern province ^43^. This variability between personal consumption and customary exchange can be explained by the annual variability of customary ceremonies from one year to the next. Indeed, when households have weddings planned for the following year, the quantities of tubers planted and harvested are greater in order to satisfy customs. The split between personal consumption and gifts may also vary according to the number of mourning events in the year. The situation is not so mechanical, however: symbolic tubers that were not exchanged during these mourning ceremonies, which cannot be predicted, were then switched to consumption.

**Figure 3.**
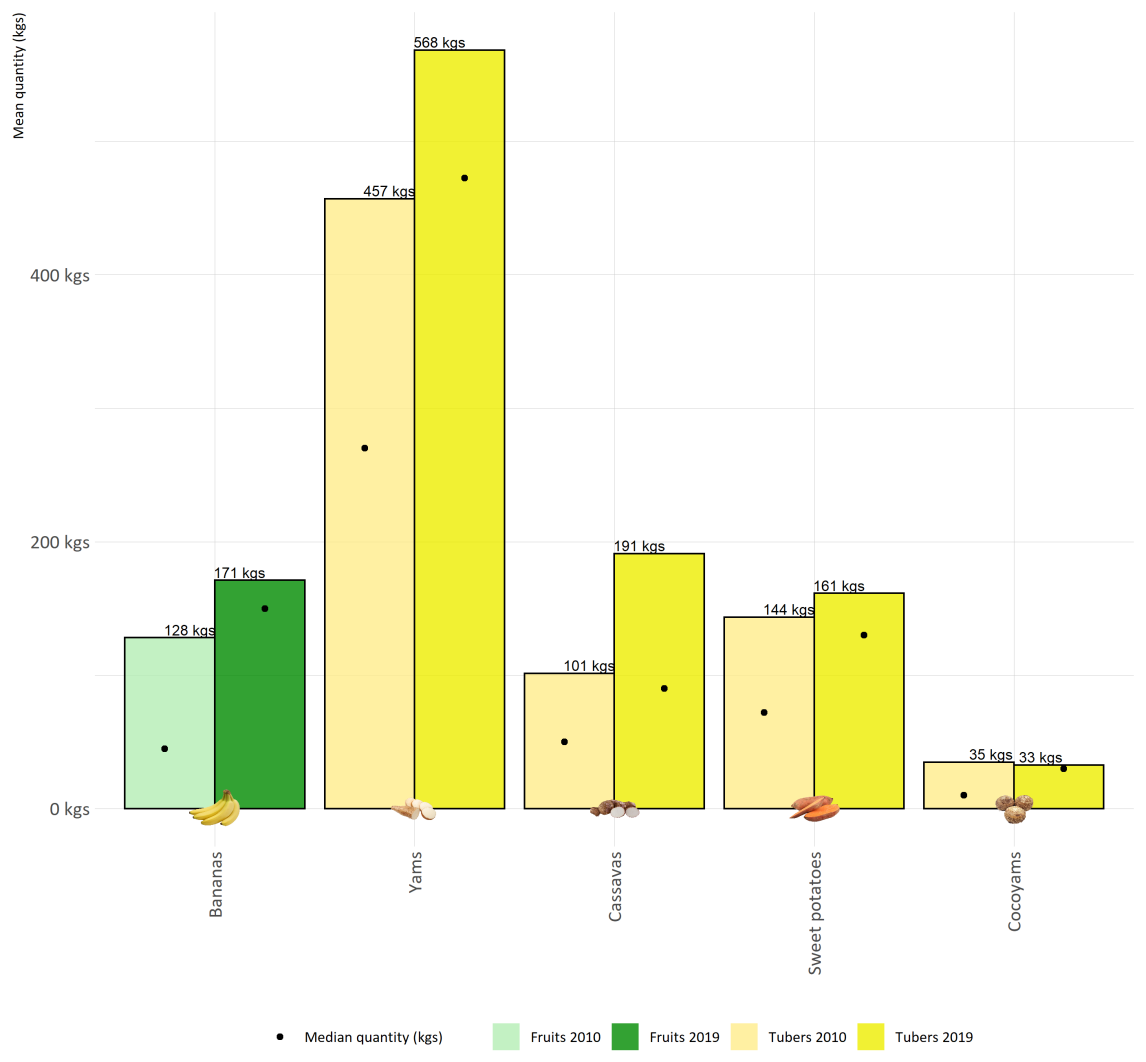
Comparison of the average quantities of the main agricultural products planted on Lifou by households in 2010 and 2019.

Indeed, Figure 3 shows the stability of tuber production at the household level. This figure is a very efficient illustration of the symbolic and cultural dimension of the Melanesian society and lifestyle. Melanesian people continue to grow yams, at least a minimal quantity, around 400 plants/year, but for their symbolic dimension.

### Relationships between traditional food consumption and family production in adults

**Figure 4.**
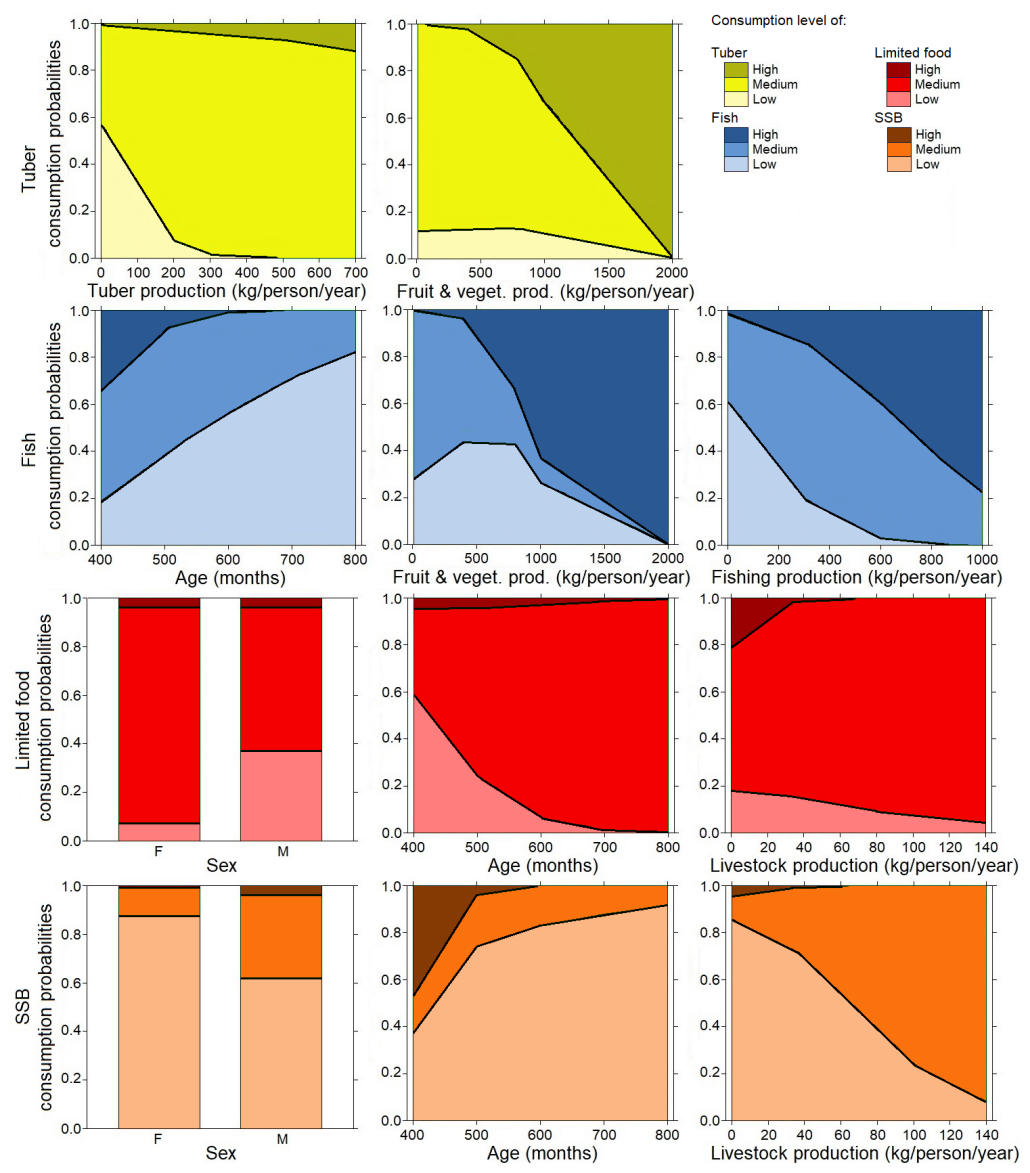
Multinominal analysis in adults: consumption of tubers (yellow color), fish (blue color), and limited drinks: sugar-sweetened beverages (SSB) (orange color) and limited foods (red color) explained by weighted agricultural variables (surface production, tuber production, weighted fish, off-farm gains), socioeconomic variables, sex, and age. Details of the analysis are in the extended data section ^28^.

Figure 4 shows that in the adult population, consumption of tubers was positively related to the production of tubers ( *P*<0.03), and negatively to fruits and vegetables ( *P*<0.01). More surprisingly, the consumption of fruits and vegetables was not linked to a social or agricultural variable. Fish consumption was negatively linked to age ( *P*<0.04) and fruit and vegetable production ( *P*<0.002), and positively linked to fish production ( *P*<0.01). The consumption of SSBs was linked to sex (males consumed significantly more SSB than females, *P*>0.03) positively to livestock production ( *P*<0.02), and negatively to age ( *P*<0.03). The consumption of limited foods was linked to sex (male consumed significantly less SSB than females, *P*<0.02), to livestock production ( *P*<0.01), positively to age ( *P*<0.007) and positively to tuber production (P = 0.05).

### Relationships between traditional food consumption and family production in children

**Figure 5.**
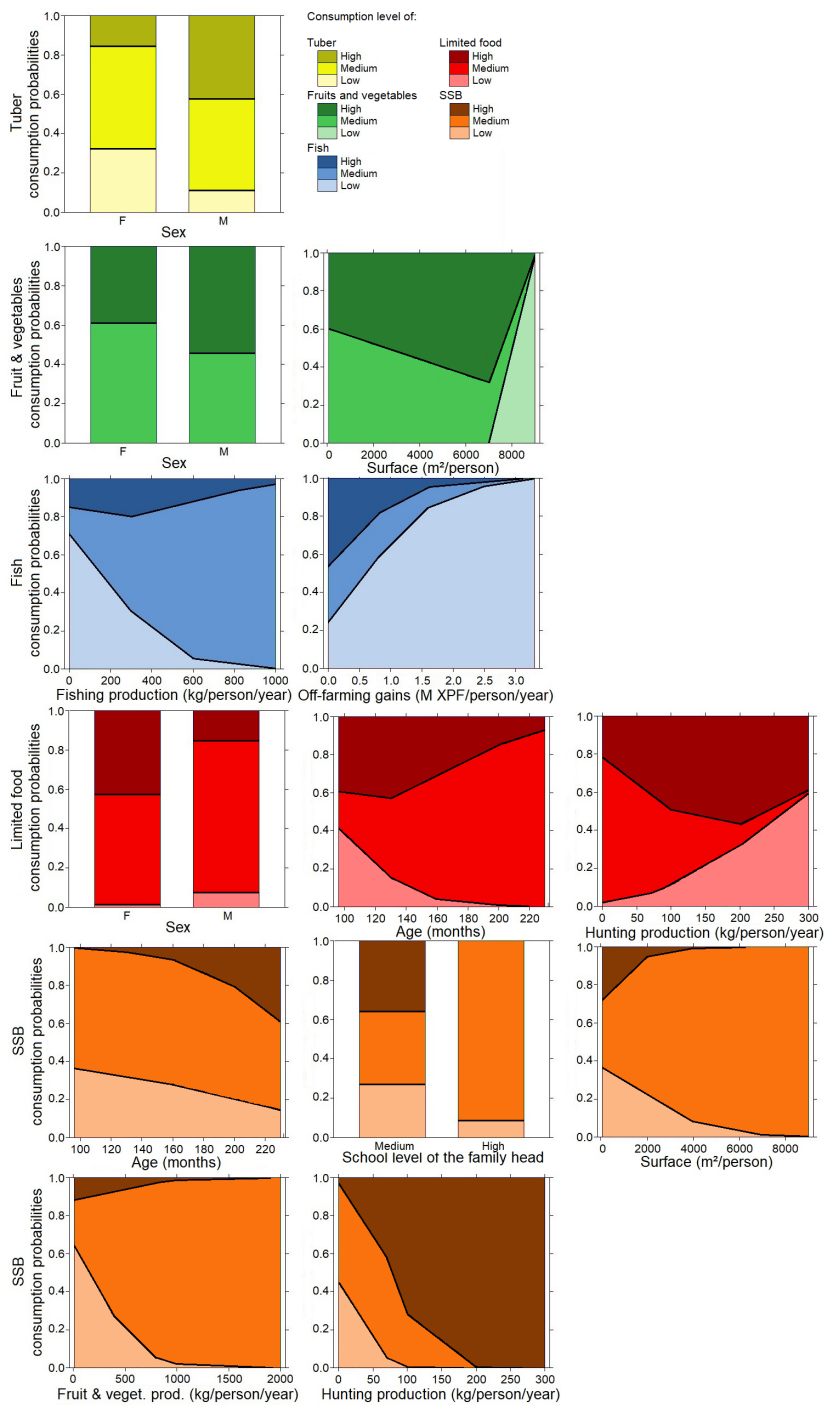
Multinominal analysis in children: consumption of tubers (green color), fruit and vegetable (green color), fish (blue color), limited drinks; sugar-sweetened beverages (SSB) (orange color) and limited food (red color) explained by weighted agricultural variables (surface production, tuber production, weighted fish, off-farm gains), socioeconomic variables, sex, and age. Details of the analysis are in the extended data section ^28^.

Figure 5 shows that in children, consumption of tubers was linked to sex (P<0.03). The consumption of fruits and vegetables was linked to sex with a higher consumption in boys ( *P*<0.001) and positively linked to fish production ( *P*<0.03), and linked to surface of the garden for agriculture with a non-monotone relation ( *P*<0.01) ^28^. Fish consumption was positively linked to fish production ( *P*<0.03) and negatively linked to off-farm gains ( *P*<0.03). The consumption of SSBs was positively linked to age ( *P*>0.04), surface of the garden for agriculture ( *P*<0.03), production of fruits and vegetables ( *P*<0.02) and hunted products ( *P*<0.001). The consumption of SSBs is also linked to the educational level of the head of the family ( *P*<0.02). The consumption of limited food was linked to sex (girls consumed significantly more limited food than boys *P*<0.01), and positively to age ( *P*>0.01) and to hunting production ( *P*>0.04).

### Relationships between agriculture and physical activity in adults

The models to explain MVPA, sedentary behavior, and international recommendations for PA did not find significant relationships with agricultural variables in adults. However, in one of the models, light PA was significantly related to age ( *P*<0.01).

### Relationships between agriculture and physical activity in children

The model to explain MVPA was significantly linked to sex (male, *P*<0.001), age ( *P*<0.04), and family production of tubers ( *P*<0.02). The model to explain light PA was significantly related to age ( *P*<0.01) and the surface used for farming ( *P*<0.02). The model to explain sedentary behavior did not find any significant relationships. The model to explain the international recommendation for PA per day was significant in the children and showed significant links between sex (male, *P*<0.01) and area of the garden used for agriculture ( *P*<0.02).

### Selected variables and logistic analysis for anthropometry, diet, physical activity and family farming in adults

The general model showed that consumption of fruits, cheese, red meat, and pig were significantly related to overweight and/or obesity. Red meat was positively related to overweight and obesity (OR=7778.73, *P*<0.043), and pig consumption was significantly inversely related to overweight and obesity (OR=2.64 E-4, *P*<0.014).

### Selected variables and logistic analysis for anthropometry, diet, physical activity and family farming in children

The general model showed that in children, fruit and vegetable production was positively linked to overweight and obesity (OR=1.01, *P*=0.013) and MVPA time was negatively linked to overweight and obesity (OR = 7.39 E-3, *P*=0.030). For children whose household head had a low or medium educational level, hunting production was positively linked to overweight and obesity, while the relationship was the reverse but weak for children whose household head had a high educational level. In girls, an average consumption of potato chips was positively associated with overweight and obesity, while in boys, the relationship appeared to be the opposite. Sedentary activity time was positively associated with overweight and obesity in girls and negatively in boys.

## Discussion

The main result of this study was that family farming production modestly contributed to healthy diets and active lifestyles for family farmers on Lifou Island. Moreover, the drivers for obesity in this rural community were diet in adults, whereas socioeconomic status and physical activity played an important role in as drivers for being overweight and obesity in children. These differences in lifestyle behaviors within families suggest an intergenerational transition in cultural practices.

### Family farming production, diet, physical activity, and obesity

We observed that family farming production of fruits and vegetables, animals, and fishing were mainly oriented toward personal consumption and exchanges rather than selling ( Table 3). Indeed, tribal family farming contributes directly to the economy and food security ^10^. Some production was destined for gift-giving and contributed to socialization (strengthening social and family ties through daily donations of plant production and mutual aid in the fields) and culture (maintaining customary traditions and donations) ^10^. The main differences compared to the last New Caledonian survey done in the three provinces were related to hunting and can be explained by the absence of deer on the island. Our results showed both a lower overall fruit and vegetable production and a trend toward reducing exchanges to the benefit of personal consumption ^24^. This is probably due to the sampling process: the households surveyed in 2019 were relatively young with children in secondary school and slightly higher incomes ( Table 3). These households were likely to be slightly less agriculturally oriented than if we had sampled from the general tribal population of Lifou. In addition, in terms of crop production, we need to take into account the variability of yields, which may have been somewhat lower due to several years of drought between 2015 and 2019. Thus, if we look at the average quantities planted, the figures are relatively stable between the two survey rounds (2010 and 2019). Indeed, Figure 3 is a very efficient illustration of the symbolic and cultural dimension of the Melanesian society and lifestyle. Melanesians continue to grow yams, at least a minimal quantity and around 400 plants/year for their symbolic dimension. Nevertheless, these cultural and traditional activities are not static but are changing, sometimes to the benefit of modern activities ^10, 18^ in which both parents and children are implicated, even in rural areas. We thus assume that we are seeing a transition in cultural practices.

The rapid economic growth that New Caledonia has experienced since the 1990s has led to the spread of wage employment in many tribes and has favored the monetarization of Kanak society. In 2010, 60% of household monetary income came from wages ^11^. Despite the large disparities between rural and urban areas ^5, 41, 42^, this gives families the purchasing power necessary to change their habits even though traditional and cultural practices stay in place at home. Moreover, Lifou Island has specific features that cannot be ignored. Lifou supports its double insularity and concentrates employment opportunities in the public and private sectors. In terms of consumption, everyday consumer goods and manufactured goods are transported by sea freight from the main island and/or are imported from overseas after a stopover on the main island. Indeed, all products not manufactured or produced on Lifou Island come by sea trade from the main island and/or are imported from overseas after a stop in the main island. This strongly increases the cost and availability of imported products that are often essential not only for everyday consumption, but also for economic development, even for agricultural development. Importing agricultural equipment (power tillers, irrigation systems, etc.) is costly and complex, with limited repair possibilities due to the lack of mechanical parts and skills.

Concerning diet, the 40 families surveyed reflect regional trends: diets on Lifou are moving away from traditional tropical tuber-based diets and increasingly include processed and imported products: rice, noodles, canned goods, frozen meat and chicken, temperate fruits and vegetables (apples, pears, garlic, onions, carrots), coffee, sugar, and oil, as well as a range of sweet and salty products. Although they may maintain their own consumption habits, households are becoming increasingly dependent on shops and the cash economy to meet their food needs. Figure 2 shows the respective consumption frequencies for pasta, rice and tropical tubers. This probably illustrates the final phase in the gradual transformation of the eating habits of Lifou families.

Tubers, which were until recently the basis of the Kanak and Oceanian diet, seem to be decreasing in importance in comparison with imported products such as pasta or rice. The consumption of tubers is not disappearing but, probably because of the long preparation time, it is becoming less frequent, while most of the surveyed households consume pasta or rice on a daily basis. These are relatively simple foods that are quick to prepare and remain cheap even though they are purchased. The most worrisome aspect of Figure 5 is the generational effect, since fewer children eat tubers several times a week (less than 1/2, compared with 2/3 of adults) and more than 7% say they never eat them. At the same time, we observed the high consumption of limited food and drinks in both adults and children, with a more pronounced consumption of limited food and drinks by girls regarding the international recommendations ( Table 2) ^5^. Similar observations were made in a larger group of Melanesian adolescent girls ^17, 18^ and confirm this trend. These results showed a juxtaposition of traditional food with highly processed food and drinks, with both contributing to “food over-abundance for meals” ^44, 45^. We might also assume that we are at a cross-point of malnutrition due to this superposition of traditional and processed foods in the daily diet of Lifou Islanders. Indeed, both high quantities of food intake and the highly caloric diet (mainly in processed food and drinks) contribute to future chronic diseases ^2, 17^.

**Table 2.** Descriptive data for the 142 participants including food frequency consumption of traditional food (tubers, vegetables, legumes and fruits, fish, and water) and physical activity in terms of duration and intensity (sedentary, light, and MVPA). In addition, weight status parameters (height, weight, BMI, and weight status) are presented. All variables are presented for parents and children regarding sex in each category, regarding international recommendations of WHO for physical activity ^54^, and regarding the Pacific Community guidelines for diet ^5^.

			Parents			Children		
		International recommendations	F & M	F	M	F & M	F	M
	Age [years]		43.97 ± 6.57	41.95 ± 5.81	46.24 ± 6.71	13.33 ± 2.45	13.67 ± 2.24	12.97 ± 2.64
Traditional food	Tubers [unit/day]		2.37 ± 1.73	2.47 ± 1.88	2.08 ± 1.38	3.13 ± 2.13	2.67 ± 1.91	3.67 ± 2.25
	Fruit and vegetables production [unit/day]	≥5	30.31 ± 14.41	31.78 ± 15.22	27.45 ± 12.14	38.97 ± 14.81	38.00 ± 15.04	42.21 ± 15.14
	Fish [unit/day]	>1.5	1.75 ± 1.33	1.72 ± 1.37	1.68 ± 1.17	2.05 ± 1.69	2.00 ± 1.77	1.95 ± 1.60
	Water [unit/day]	≥7	3.36 ± 1.03	3.23 ± 1.18	3.51 ± 0.84	3 ± 1.08	2.71 ± 1.15	3.29 ± 0.94
Processed food and drinks	Limited foods [unit/day]	<1	3.19 ± 1.78	3.39 ± 1.81	2.98 ± 1.75	4.46 ± 2.64	5.47 ± 2.8	3.45 ± 2.04
	Limited beverages [unit/day]	<1	0.46 ± 0.81	0.33 ± 0.65	0.6 ± 0.94	0.95 ± 1.09	1.13 ± 1.29	0.76 ± 0.83
Duration and intensity of daily physical activity	Sedentary [min/day]	-	704 ± 128	703 ± 113	706 ± 144	672 ± 82	670 ± 69	673 ± 97
	Light [min/day]	-	88 ± 36	81 ± 31	95 ± 41	47 ± 7	49 ± 13	45 ± 20
	Moderate-to-vigorous [min/day]	≥ 60 min	17 ± 9	18 ± 8	16 ± 10	46 ± 22	36 ± 13	58 ± 24
	MVPA number (%)	Children: MVPA ≥ 3600s/day; Adults: MVPA ≥ 965s/day	34 (48.57%)	19 (52.78%)	15 (44.12%)	14 (22.22%)	1 (2.94%)	13 (44.83%)
Weight status	Height [cm]		165.9 ± 8.6	159.6± 4.1	173.1 ± 6.5	157.8 ± 12.9	158.1 ± 9.3	157.3 ± 16
	Weight [kg]		87.3 ± 20.31	78.26 ± 18.14	97.44 ± 17.85	54.11 ± 16.86	56.48 ± 15.33	51.53 ± 18.27
	BMI [kg/m²]	18.5 <=BMI <= 25	31.55 ± 6.39	30.64 ± 6.56	32.57 ± 6.12	21.36 ± 4.95	22.41 ± 5.33	20.21 ± 4.28
	IOTF z-score		-	-	-	0.63 ± 1.04	0.78 ± 0.98	0.47 ± 1.09
Weight Status (%) BMI for parents IOTF for kids	Underweight	BMI<18.5 (adult)	0%	0%	0%	3.1%	0%	6.2%
	Normal	18.5<=BMI<25 (adult)	14.3%	21.6%	6.1%	67.2%	65.6%	68.7%
	Overweight	BMI>=25 (adult)	25.7%	24.3%	27.3%	22%%	25%	18.7%
	Obese	BMI>=30 (adult)	60%	54%	66.7%	7.8%	9.4%	6.2%

**Table 3.** Family farming productions and their destinations: sale, personal consumption, or exchange for: (1) the last national study on Melanesian family farming in New Caledonia concerning Lifou Island (IAC, 2010), and (2) for the present study in 2019. Concerning production, tubers (taro, yam, cassava, etc.), fruits and vegetables (avocado, star fruit, lemon, salad, etc.), and total crop production are presented. The same descriptive data can be found for hunting and fishing. Garden soil was analyzed by its surface.

	Details of the category	Production on customary land and destination in 2010 (IAC, 2014)	Production on customary land and destination on Lifou for the study (2019)
Family	Family members (n)	4.09 ± 2.65	5.43 ± 1.08
	Active members (15 to 65 years old)		3.18 ± 1.13
School level	No diploma (n)		1
	Primary school diploma (n)		2
	Secondary school diploma (n)		25
	Degrees or higher diploma (n)		12
Plant production	Total (kg/yr)	3903±4693	3181.28 ± 2007.46
	Sold (kg/yr)	343±967	163.64 ± 444.39
	Exchange (kg/yr)	852±1522	336.36 ± 284.66
	Consumed (kg/yr)	971±1085	1697.48 ± 895.84
Animal production	Total (kg/yr)	127±752	135.05 ± 175.55
	Sold (kg/yr)	97±649	14.7 ± 65.59
	Exchange (kg/yr)	121±219	98.7 ± 121.63
	Consumed (kg/yr)	59±116	21.65 ± 72.94
Hunting	Total (kg/yr)	318±495	139.93 ± 296.05
	Sold (kg/yr)	8±63	1.25 ± 7.91
	Exchange (kg/yr)	137±285	24.4 ± 71.36
	Consumed (kg/yr)	172±267	114.28 ± 274.98
Fishing	Total (kg/yr)	670±1115	631.63 ± 1099.97
	Sold (kg/yr)	168±572	8.38 ± 28.16
	Exchange (kg/yr)	120±335	41.46 ± 154.49
	Consumed (kg/yr)	379±575	581.79 ± 1055.33
Off-farm gains	Gains (M XPF)	5473.56±5412.93	4441.65 ± 3143.08

In this rural community, only 48% of the adults and 22% of children (with significantly lower values for girls compared to boys) met the international recommendations for MVPA ( Table 2). The distribution of PA in adults or children showed that most daily activities are sedentary or qualify as only light PA ( Table 2). These data confirm previous observations reported in adolescents via accelerometry ^22^. Physical activity levels in rural agricultural areas and livelihood activities are known to be higher compared with sedentary urban living, but these activities predominantly involve light and moderate levels of activity rather than moderate-to-vigorous activity ^46^. This is the case of family farmers in the PICTs, where rurality plays an important role in the societies. Very recently, studies have pointed to the important place of PA in small-scale farmers in rural areas of India, Nepal, and Ghana ^47, 48^. In Bolivia, the Tsimane, who are forager-horticulturalists of Amazonian Bolivia living traditional lifestyles, reportedly engage in vigorous daily activity that protects against noncommunicable diseases and show minimal heart disease and diabetes ^49^. Throughout the year, physical activity remains high and Tsimane men show higher physical activity levels than women at all ages. In our study, the overall diet and PA observed in the adults in our study showed the superposition of traditional and modern diets, with high consumption of limited food and drinks, although 48% of them reached the international recommendations for PA. Meanwhile, the children showed a similar diet pattern but less than 22% of them reached the PA recommendations. This suggests a less active life, with parents doing the family farming and little involvement from the children.

We observed that more than 85% of the adults and more than 30% of the children were overweight or obese. For this reason, a deeper analysis of the specific relationships between family farming, diet, and physical activity are needed to better understand this intergenerational lifestyle transition.

### Relationships between family farming and traditional diet

In adults, fishing activities were found to make a large contribution to the traditional diet ( Figure 4), with links observed between consumption of tubers and fishing activity in the adult group. This result confirms the place of traditional tribal and cultural activities that are supported by adults. We also observed the high consumption of limited foods, mainly by women and older adults. This has already been observed in other contexts and might be explained by who does the cooking, which is often women in Pacific communities ^50, 51^; the time dedicated to cooking in working families ^52^, which is decreasing with the spread of wage employment; and having the money to buy imported food, which can save time in the kitchen and satisfy the tastes of children used to collective catering. Eating limited foods can also be seen as a way of diversifying menus, sometimes wrongly. Last, but not least, for the elderly in the community, it can also be a way of ostensibly showing their personal purchasing power ^15, 16^. These many factors thus contribute to the co-existence of traditional and modern diets.

In children, the consumption of tubers, fruits, and vegetables was greater in boys and increased with the surface of family farming production ( Figure 5). This can be explained by the place of boys in family tasks and the nutritional needs of active children with the consumption of energetic products (tubers). Net fishing, sparrowhawk fishing, underwater hunting, and diving are part of the tasks assigned to boys in the community, while fishing from the shore and angling are more feminine practices ^53^, and this would explain why fish consumption in boys was likely to be linked to the fish production of the family. Another interesting point was that limited drinks consumption was likely to be related to the educational level of the family head. A possible explanation may be that having money to buy food may well be a way to ostensibly show the personal earning power of families with high levels of education and money income in the community ^18, 19^.

### Relationships between family farming and energy expenditure

We observed that in the adult family farmers, light physical activity increased with age, indicating that older generations spend more time in family activities and gardening. In children, MVPA was more likely to be high in boys, increasing with age and related to the family production of tubers. Light PA was more likely to be high with age and with the surface of the garden exploited by the family. In addition, meeting the international recommendations for PA per day was likely to be high in boys and with the surface of the family garden. Combining energy expenditure data from accelerometry devices with time-use data provides a window into rural agricultural and livelihood activities that had once been unavailable ^46^, as it links how food is produced with nutritional aspects ^46, 55^. This link has often been neglected, even though most smallholder farmers, especially in developing countries, rely on hand tools for farming ^56^. A similar traditional approach to family farming without mechanization persists in the Pacific region and on Lifou Island, even though income per capita is above that of developing countries.

### What are the drivers for obesity in family farming production, diet, and physical activity?

The general model showed that diet and especially fruits, cheese, red meat, and pig were the main drivers of adult overweight and/or obesity (see extended data - supplementary multinomal analysis data file) ^28^. The protein leverage hypothesis holds that dietary protein is positively related to overweight and obesity ^57^. There is now strong evidence that excess protein intakes are associated with lifestyles with low physical activity and accelerated aging, which would seem to be the perfect combination to drive to obesity and cardiometabolic diseases ^58^ and would explain the main observation in this general model. The general model for children underlined that MVPA was the main driver to prevent the development of obesity (see extended data - supplementary multinomal analysis data file) ^28^. Indeed, physical activity in children and adolescents is associated with improved physical, mental, and cognitive health outcomes ^54^. The evidence shows clearly that increased time in aerobic MVPA increases cardiorespiratory fitness and that increased muscle-strengthening activities increase muscle fitness, with some evidence showing incremental benefits of doing both ^54^. In the present study, this was reinforced by the role of the family environment since obesity was inversely related to the level of education of the family head. In transitioning countries, a high educational level plays an important role in preventing overweight and obesity in households and contributes to global development ^5^. Moreover, we observed that limited foods and high sedentary time in girls were the drivers to overweight and obesity. This has frequently been observed to be related to adverse health outcomes ^54^. Indeed, the association between sedentary behavior and adverse health outcomes is generally stronger with high media use and associated snacking, with both contributing to total sedentary time ^54^. 

This interdisciplinary study, which included researchers in the exercise and health sciences, nutrition, geography, agriculture, and informatics disciplines, provided data that enriches our understanding of the complex relationships between family farm production, traditional food in diet, physical activity, and obesity. The study enabled us to analyze the data at individual and family scales to make sense of our results in such a way that we were able to gain insight into family farming, diet, physical activity, and a health ecosystem at the scale of a community.

### Limitations

Although our sample was small, which calls for caution in generalizing to the whole rural community of Pacific Islands Countries and Territories, the Melanesian community is well represented among the Lifou family farmers, and the amount and quality of data collected per household and individual brought important information.

As described in the method section, for accurately comparing individual data, we converted “farming data” according to the number of people in the household. The proportion of adults/children was not taken into account because in the questionnaire the family members number is the only information we get without distinction between the number of adults and children. In addition, children’s consumption is very variable according to age. Usually, a child before adolescence eats a smaller quantity of food than an adult whereas during adolescence a higher consumption can be observed when compared to adulthood. As a consequence, discriminating production and consumption of production of a household according to, not only its size, but according to its composition too, would raise complex issues in analyses that are beyond the scope of the current study. Finally, we used the ratio computation according to the household size in analyses as a reasonable alternative to avoid an imbalance between individuals belonging to large families and those belonging to small families when analyzing production and consumption of production.

While the data from accelerometry did not permit us to identify the time and intensity dedicated to family farming tasks, we extracted data including these daily tasks plus other daily activities for a continuous period of 24 hours per day over seven days. We assume that the quantity and quality of the data added to the robust data mining analysis, which brought reliable information on PA.

## Conclusion

We conclude that family farming makes a modest contribution to the diet and daily activity of the family farmers on Lifou Island. Today, family farming is still a vital activity, though more for its social and cultural dimensions than for subsistence. The differences in lifestyle behaviors within families suggest an intergenerational transition in cultural practices. For example, we found that the drivers of obesity in adults were related to diet, whereas the socioeconomic status of the parents and MVPA played an important role in preventing being overweight and obesity in children. So, reinforcing the vivacity of cultural practices linked to family farming across the generations should be accompanied in the development of civil and customary policies of Lifou island.

Future studies should take a broader approach to family farming lifestyle and health in Melanesia, including Vanuatu, Fiji, Solomon Island, and Papua New Guinea. In addition, the seasonality of family farming and a more in-depth analysis of diet including macro- and micro-nutrients would yield more accurate information on the intergenerational transition in cultural practices and its consequences for noncommunicable diseases. Moreover, more interdisciplinary approaches should be developed since we effectively identified adapted scales between agriculture, diet, exercise, and health by using mixed methods.

## Data Availability

Zenodo: Generational issues in linking family farming production, traditional food in diet, physical activity and obesity in Pacific Islands countries and territories: the case of the Melanesian population on Lifou Island. https://doi.org/10.5281/zenodo.5516813 ^28^. The project contains the following underlying data: -    PIURN_households_openData_2021-08-31. (CSV files containing household composition including number of persons, education, land access and ownership, monetary incomes; the quantification of agricultural production : plants, cattle, hunting, fishing, and food gathering and; the allocation of these productions in the eating and sharing (daily and ritual exchanges) habits of the families). -    PIURN_individual_openData_2021-08-31. (CSV files containing individual data of chidren and parents extracted from the food frequency questionnaire, anthropometry, and accelerometry). Data are available under the terms of the Creative Commons Zero “No rights reserved” data waiver (CC0 1.0 Public domain dedication). Zenodo: Generational issues in linking family farming production, traditional food in diet, physical activity and obesity in Pacific Islands countries and territories: the case of the Melanesian population on Lifou Island. https://doi.org/10.5281/zenodo.5516813 ^28^. This project contains the following extended data: -   Supplementary multinomal analysis data. (CSV files containing statistical analysis and tables supporting figures 4 and 5). -   Supplementary prodagri data. (CSV files containing data supporting figure 2 and 3). -   Questionnaires. (French and English version of the family farming questionnaire and food frequency 23uestionnaire used in this pilot case study). Data are available under the terms of the Creative Commons Zero "No rights reserved" data waiver (CC0 1.0 Public domain dedication).
