# Supplementary material for: Generational issues in linking family farming production, traditional food in diet, physical activity and obesity in Pacific Islands countries and territories: the case of the Melanesian population on Lifou Island
Source: Open Res Eur. 2022 Oct 11;1:135. Originally published 2021 Nov 11. [Version 2] doi: 10.12688/openreseurope.13705.2 (PMC10446099; doi:10.12688/openreseurope.13705.2)
Supplement: Peer review history

# Peer review: Reviewer response for version 2

**DOI:** 10.21956/openreseurope.16324.r30268

**Published:** 2022-10-18

**Authors:** Louisa Peralta

**Affiliations:** 1 School of Education and Social Work, Faculty of Education and Social Sciences, The University of Sydney, Sydney, NSW, Australia

Dear Editor and Author(s), 

 We appreciate your responses and changes based on the feedback/recommendations. The manuscript has been strengthened and I have no additional changes/recommendations. 

 Thank you,

 Reviewer

Is the case presented with sufficient detail to be useful for teaching or other practitioners?

Yes

Is the work clearly and accurately presented and does it cite the current literature?

Yes

If applicable, is the statistical analysis and its interpretation appropriate?

Yes

Are all the source data underlying the results available to ensure full reproducibility?

Yes

Are the conclusions drawn adequately supported by the results?

Yes

Is the background of the case’s history and progression described in sufficient detail?

Yes

Reviewer Expertise:

NA

I confirm that I have read this submission and believe that I have an appropriate level of expertise to confirm that it is of an acceptable scientific standard.

# Peer review: Reviewer response for version 1

**DOI:** 10.21956/openreseurope.14779.r28732

**Published:** 2022-04-04

**Authors:** Marta Plichta

**Affiliations:** 1 Warsaw University of Life Sciences, Warsaw, Poland

In the manuscript “Generational issues in linking family farming production, traditional food in diet, physical activity and obesity in Pacific Islands countries and territories: the case of the Melanesian population on Lifou Island” the Authors tried to determine the links between family farming, diet, physical activity and obesity in Melanesian Lifou Island families. The pilot study included forty families, including 142 adults and children, from Lifou Island.

 The manuscript provides valuable information, However, I have some questions.

 The introduction introduces the reader well to the subject of the study, pointing out problems related to modern lifestyles in the Pacific region, followed by negative consequences such as fundamental changes in eating behaviors and physical activity and major increase in the noncommunicable diseases. In this context, the Authors refer to the effect of family farming in health and the possible changes of behavior between parents and children in Melanesian population.  

 I have a question about participants. Why the Authors have chosen people who lived all year round on Lifou Island? You mean they could not leave the Island even for a short time? Explain, please. 

 The families which took part in the study were randomly selected by pulling numbers out of a box. This is important in research because random selection allows us to relate the results obtained in the sample to the general population. 

 However, I have a question about the age of children. Why have the Authors selected families among the classes in which children were 11-15 years old? Why not also younger? 

 The Authors describe step by step what they did in the study, as well as who was responsible for what and why which is important from the point of view of other researchers who would like to repeat this study.  

 The limitations of this study are that the proportion of adults, children, and adolescents was not taken into account in this calculation.  

 The results are presented clearly, which are supported by many figures and tables.

Is the case presented with sufficient detail to be useful for teaching or other practitioners?

Yes

Is the work clearly and accurately presented and does it cite the current literature?

Yes

If applicable, is the statistical analysis and its interpretation appropriate?

Yes

Are all the source data underlying the results available to ensure full reproducibility?

Yes

Are the conclusions drawn adequately supported by the results?

Yes

Is the background of the case’s history and progression described in sufficient detail?

Yes

Reviewer Expertise:

My areas of research are human nutrition, nutrition psychology, public health, eating disorders, eating behaviors.

I confirm that I have read this submission and believe that I have an appropriate level of expertise to confirm that it is of an acceptable scientific standard.

# Response

**Published:** 2022-09-07

**Authors:** olivier Galy

**Affiliations:** university of new caledonia, New Caledonia

We thank the reviewer for her appraisals. We have done our best to address the concerns and hope that the manuscript is now suitable for publication.  

*In the manuscript “Generational issues in linking family farming production, traditional food in diet, physical activity and obesity in Pacific Islands countries and territories: the case of the Melanesian population on Lifou Island” the Authors tried to determine the links between family farming, diet, physical activity and obesity in Melanesian Lifou Island families. The pilot study included forty families, including 142 adults and children, from Lifou Island.   The manuscript provides valuable information, However, I have some questions.   The introduction introduces the reader well to the subject of the study, pointing out problems related to modern lifestyles in the Pacific region, followed by negative consequences such as fundamental changes in eating behaviors and physical activity and major increase in the noncommunicable diseases. In this context, the Authors refer to the effect of family farming in health and the possible changes of behavior between parents and children in Melanesian population.*

*Q1.I have a question about participants. Why the Authors have chosen people who lived all year round on Lifou Island? You mean they could not leave the Island even for a short time? Explain, please.*

 R1: People can leave Lifou island when they want and for the duration they want. We included people who are living most of the time in Lifou island because they are the part of Lifou population that is active for farming and hunting. Another part study and/or work in mainland and go back to Lifou for short periods that does not permits to be regularly involved in family farming activities.  

*Q2:The families which took part in the study were randomly selected by pulling numbers out of a box. This is important in research because random selection allows us to relate the results obtained in the sample to the general population. However, I have a question about the age of children. Why have the Authors selected families among the classes in which children were 11-15 years old? Why not also younger?*

 R2: Studies done in nutrition usually shows that children are able to do a survey alone around 10 to 11 years old. Our group has a large expertise in experimentations in this age group, mainly in the Pacific region and native language can add a difficulty if children are youngest. That is why we choose adolescents between 11 and 15 years old.

# Peer review: Reviewer response for version 1

**DOI:** 10.21956/openreseurope.14779.r28864

**Published:** 2022-03-28

**Authors:** Louisa Peralta

**Affiliations:** 1 School of Education and Social Work, Faculty of Education and Social Sciences, The University of Sydney, Sydney, NSW, Australia

Dear Author(s),

 An interesting paper that is well-written (in most sections). Thank you for the opportunity to read through the manuscript to provide feedback and recommendations:

- Grammatical errors - Plural on end of Melanesian population (to Melanesian populations).
- Research design is not clear. Is it a pilot case study? It has been called a pilot study in the abstract and case study in the methods.
- Methods: 142 participants is substantial. Well done. What was the recruitment % (of those who you asked to participate in the study and those who consented?). What was the population % (142 participants of how many on Lifou)? 142 Melanesian families of how many in the Melanesia and the PICTs?
- Results: Were you able to map objective PA data to farming activities (regular farming activities) to get an indication of how much of the MVPA was attributed to farming and other activities?
- Discussion and conclusion: A clear statement about how this research may contribute to the development of policies on Lifou Island. What is needed to reduce the intergenerational transition?

Thank you,

 Reviewer.

Is the case presented with sufficient detail to be useful for teaching or other practitioners?

Yes

Is the work clearly and accurately presented and does it cite the current literature?

Yes

If applicable, is the statistical analysis and its interpretation appropriate?

Yes

Are all the source data underlying the results available to ensure full reproducibility?

Yes

Are the conclusions drawn adequately supported by the results?

Yes

Is the background of the case’s history and progression described in sufficient detail?

Yes

Reviewer Expertise:

Health and Physical Education, Physical Activity and Populations.

I confirm that I have read this submission and believe that I have an appropriate level of expertise to confirm that it is of an acceptable scientific standard, however I have significant reservations, as outlined above.

# Response

**Published:** 2022-09-07

**Authors:** olivier Galy

**Affiliations:** university of new caledonia, New Caledonia

We thank the reviewer for her appraisals. We have done our best to address the concerns and hope that the manuscript is now suitable for publication.   

*Q1.Grammatical errors - Plural on end of Melanesian population (to Melanesian populations).*

 R1: An harmonisation had been done in the manuscript to keep “the Melanesian population” *.*

*Q2. Research design is not clear. Is it a pilot case study? It has been called a pilot study in the abstract and case study in the methods.*

 R2: Effectively, the term “pilot case study” is more appropriate. This has been changed throughout the manuscript.

*Q3.Methods: 142 participants is substantial. Well done. What was the recruitment % (of those who you asked to participate in the study and those who consented?). What was the population % (142 participants of how many on Lifou)? 142 Melanesian families of how many in the Melanesia and the PICTs?*

 R3: We recruited 48 families and 40 families accepted to participate and compose the sampling of 142 participants. This is detailed in the “data collection participants” section.

*Q4.Results: Were you able to map objective PA data to farming activities (regular farming activities) to get an indication of how much of the MVPA was attributed to farming and other activities?*

 R4: Unfortunately, we were not able to map different daily activities in this pilot case study. This is planned later in the next works. This has been addressed in the manuscript, please see in “discussion section” in the last paragraph.

*Q5.Discussion and conclusion: A clear statement about how this research may contribute to the development of policies on Lifou Island. What is needed to reduce the intergenerational transition?*

 R5: This is now addressed in the conclusion.
